# Effects of rearing host species on the host-feeding capacity and parasitism of the whitefly parasitoid *Encarsia formosa*

**DOI:** 10.1093/jis/14.1.118

**Published:** 2014-09-01

**Authors:** Peng Dai, Changchun Ruan, Liansheng Zang, Fanghao Wan, Linzhou Liu

**Affiliations:** 1 Engineering Research Center of Natural Enemy Insects, Institute of Biological Control, Jilin Agricultural University, Changchun, Jilin 130118, China; 2 Key Laboratory for Biology of Plant Disease and Insect Pests, Institute of Plant Protection, Chinese Academy of Agricultural Sciences, Beijing 100094, China

**Keywords:** *Bemisia tabaci*, *Trialeurodes vaporariorum*, host feeding, mass rearing, biological control

## Abstract

Parasitoids of the *Encarsia* genus (Hymenoptera: Aphelinidae) are important biological control agents against whiteflies. Some of the species in this genus not only parasitize their hosts, but also kill them through host feeding. The whitefly parasitoid, *Encarsia formosa* Gahan, was examined to determine whether the rearing host species affects its subsequent host-feeding capacity and parasitism. *E. formosa* wasps were reared on *Trialeurodes vaporariorum* (Westwood) (Hemiptera: Aleyrodidae) and *Bemisia tabaci* (Gennadius) ‘Q’, and their subsequent host-feeding capacity and parasitism of *T. vaporariorum* and *B. tabaci* were examined. *E. formosa* reared on *T. vaporariorum* were significantly larger in body size than those reared on *B. tabaci* , but these wasps killed a similar number of whitefly nymphs by host feeding when they attacked the same host species on which they were reared. Regardless of the species on which it was reared, *E. formosa* fed significantly more on the *B. tabaci* nymphs than on the *T. vaporariorum* nymphs. The number of whitefly nymphs parasitized by *E. formosa* differed between the wasps reared on *T. vaporariorum* and those reared on *B. tabaci* depending on which whitefly species was offered as a host. In addition, the wasps reared on *T. vaporariorum* parasitized significantly more on *T. vaporariorum* than those reared on *B. tabaci* . The wasps reared on *B. tabaci* , however, parasitized similar numbers of whiteflies of both host species. The results indicated that the host-feeding capacity of *E. formosa* was affected more by the host species attacked than by the rearing host species, but the parasitism was affected by the host species attacked and the rearing host species. Generally, *E. formosa* reared on *T. vaporariorum* killed more *T. vaporariorum* nymphs by parasitism and host feeding than those reared on *B. tabaci.* Additionally, a similar number of *B. tabaci* nymphs were killed by parasitism and host feeding regardless of the rearing host species. Currently coexistence of *B. tabaci* and *T. vaporariorum* on vegetable crops usually occurs in some areas; our results may provide helpful information on using mass-reared parasitoids against mixed whitefly infestations in biological control programs.

## Introduction

Some species of insect parasitoids, particularly species of the order Hymenoptera, parasitize or deposit their eggs in their hosts and feed on the hosts (host feeding), which often results in host mortality ( Jervis and Kidd 1986 , Heimpel and Collier 1996 ). Host feeding mainly involves the consumption of host fluids that exude from the ovipositor insertion sites. This behavior has been observed in more than 140 species belonging to 17 Hymenoptera families ( Jervis and Kidd 1986 ). In addition to killing their hosts, the parasitoids obtain essential nutrients from their hosts, which results in increased egg production and/or prolonged longevity ( Giron et al. 2004 , Burger et al. 2005 , Ueno and Ueno 2007 ). In theory ( Yamamura and Yano 1988 ) and in practice ( Jervis et al. 1996 ), parasitoid species with host-feeding habits are promising agents for the effective biological control of pest insects.

*Encarsia formosa* Gahan (Hymenoptera: Aphelinidae), one of the most successfully commercialised natural enemies used for the biological control of insect pests, has been used to control the whiteflies *Trialeurodes vaporariorum* (Westwood) (Hemiptera: Aleyrodidae) and *Bemisia tabaci* (Gennadius) in many countries throughout the world (Hoddle et al. 1998). This parasitoid is a typical non-concurrent destructive host feeder that uses different host individuals for oviposition and host feeding ( Van Roermund and Van Lenteren 1997 , Zang and Liu 2008 ). *E. formosa* feeds on all nymphal instars and pupae of *T. vaporariorum* ( van Alpen et al. 1976 ) and *B. tabaci* ( Zang and Liu 2008 ) by probing the nymphs or pupae with the ovipositor for up to 6 min and then feeding from the wounds, which the wasp may enlarge with its mandibles ( van Alpen et al. 1976 ). This probing followed by feeding kills whitefly nymphs.

We previously reported that *E. formosa* could consume approximately 10 first-second instars or five third-fourth instars of *B. tabaci* in 48 h ( Zang and Liu 2008 ). A more recent study indicated that their capacity for host feeding on whitefly nymphs can be improved by food deprivation before release ( Zang and Liu 2010 ).

The effects of the rearing host species on reproductive performance have been studied for numerous *Trichogramma* spp., ( Corrigan and Laing 1994 ) and *E. formosa* ( Henter and van Lenteren 1996 , Hoffmann et al. 2001 , Ozder and Kara 2010 ). To our knowledge, however, the effects of the rearing host species on host feeding in parasitoids have not been studied. *E. formosa* attacks at least 15 hosts in eight aleyrodid genera ( Hoddle et al. 1998 ), and *T. vaporariorum, Trialeurodes ricini* Mistra and *B. tabaci* are all used as rearing hosts for *E. formosa* ( Scopes and Biggerstaff 1971 , Shishehbor and Brennan 1995, Hoddle et al. 1998 ). In this study, we investigated how the rearing host species, either *T. vaporariorum* or *B. tabaci* ‘Q’, affects the host-feeding capacity and parasitism of *E. formosa* on these same hosts. The results from this study will help to explore the biological potential of natural enemies to improve their use in biological control programs.

## Materials and Methods

### Insects and host plants

*E. formosa* wasps were originally supplied by Koppert Biological Systems (The Netherlands) in August 2008. Following procurement, the parasitoids were continuously kept in an insectary (26 ± 2°C, 60 ± 5 % RH, with a *porariorum* and *B. tabaci* ‘Q’ hosts that were maintained on potted tomato plants *(Solanum lycopersicum* L. cv. ‘Ruiqi I’ (Solanales: Solanaceae)) in separate screened cages (60 × 60 ****x**** 60 cm). The biotype ‘Q’ is a cryptic species of *B. tabaci* ( De Barro et al. 2011 ). We retained the existing biotype terminology to avoid confusion and to ensure that this study can be connected with other reports. The two host-parasitoid populations were maintained for about six generations before the experiments. Voucher specimens of the parasitoids and whiteflies have been deposited in the Insect Collection, Institute of Biological Control, Jilin Agricultural University at Changchun, China.

The tomato plant was used as the host plant for both of the whitefly species. The plants were individually grown in 15-cm plastic pots filled with growth medium (Tianyun Fertilizer, China) and enclosed in whitefly-proof screened cages. Plants with six fully-extended leaves were used in the experiments.

### Host feeding and parasitism by *E. formosa* reared on different host species

Tomato leaves with nearly emerged parasitoids were placed in large Petri dishes (15.0 cm diam and 1.5 cm deep) and were monitored every 10 min. A newly emerged *E. formosa* (< 3 h) reared on *T. vaporariorum* or *B. tabaci* was introduced onto a detached tomato leaf with 40 third instars of *T. vaporariorum* or *B. tabaci.* The third instars of *T. vaporariorum* or *B. tabaci* were selected as attacked hosts because they are preferred for parasitism and host feeding by *E. formosa* ( Nell et al. 1976 , Zang and Liu 2008 ).

**Figure 1.**
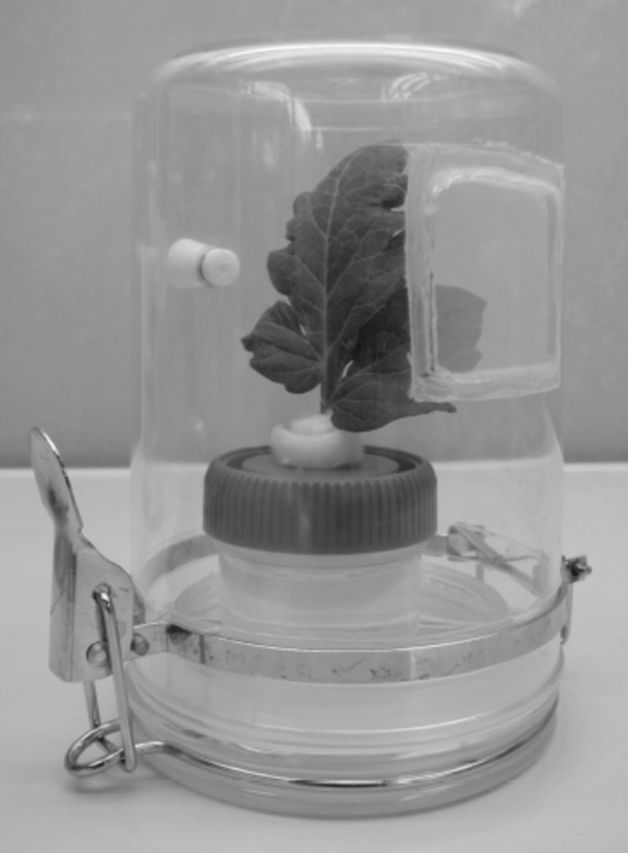
A clear plastic cell containing whitefly nymphs on a water-cultured tomato leaf used for the evaluation of host feeding and parasitism by whitefly parasitoids, *Encarsia formosa* .

The following procedures were used to obtain the desired stage of the hosts. Thirty unsexed adults of *B. tabaci* or 40 adults of *T. vaporariorum* were introduced onto the lower surface of the leaf of a potted tomato plant in a clip cage (4.0 cm diam) for oviposition for 12 h. The nymphs were then monitored daily until they developed into third instars. Forty nymphs of the desired stage were used on each leaf, and extra whitefly nymphs were removed under a binocular stereoscopic microscope using an insect pin. The petiole end of the detached tomato leaf with whitefly nymphs was wrapped with cotton, inserted into a 60-mL cup full of water, and then placed in a clear plastic cup (10 cm diam, 15 cm deep) with a ventilation hole (4 × 4 cm) and covered with a 100-mesh polyethylene screen. This clear plastic cell was used to evaluate the host feeding and parasitism by *E. formosa* on the whitefly nymphs ( Fig. 1 ). After a 48-h exposure time, the survival of the introduced wasps in each treatment was determined, and they were subsequently removed.

All of the experiments were conducted in an insectary under the above-mentioned conditions. The host mortality caused by host feeding and parasitism was examined under a stereoscopic microscope 8 d after the removal of parasitoids. The determination of whitefly nymph mortality caused by host feeding and parasitism was carried out as described by Zang and Liu (2008) . Each treatment was replicated 25 times.

### Body size of *E. formosa* reared on different host species

Three females of *E. formosa* were introduced into the clip cage on a tomato leaf infested with -50 fourth instars of *B. tabaci* ‘Q’ or *T. vaporariorum* for 12 h. The parasitoid adults were then aspirated out, the clip cage was removed, and the plant was maintained undisturbed in a screen cage (60 × 60 × 60 cm). The development of the parasitoids was monitored daily until the adults emerged. These adults, reared on either *B. tabaci* or *T. vaporariorum* (<12 h), were collected in 10-mL glass vials and placed at **—** 20°C for 2 h. The head width, body length, and hind tibia length for each individual parasitoid were measured, as described by Roskam et al. (1996) . We observed 50 *E. formosa* individuals each reared on *T. vaporariorum* and *B. tabaci* (100 in all).

### Statistical analysis

A two-factor analysis of variance (ANOVA) was performed to compare *E. formosa* reared on different host species with respect to their host-feeding capacity and parasitism on different host species. The two factors analysed were the rearing host species (two levels) and the attacked host species (two levels). The means were separated with Tukey HSD test at *P* < 0.05. A Student *t* -test was used to analyze the body size of *E. formosa* reared on *T. vaporariorum* and *B. tabaci* . All of the statistical analyses were performed using the DPS (Data Processing System) software ( Tang and Zhang 2012 ).

## Results

### Whitefly nymphs killed by *E. formosa* reared on different host species Host feeding.

**Figure 2.**
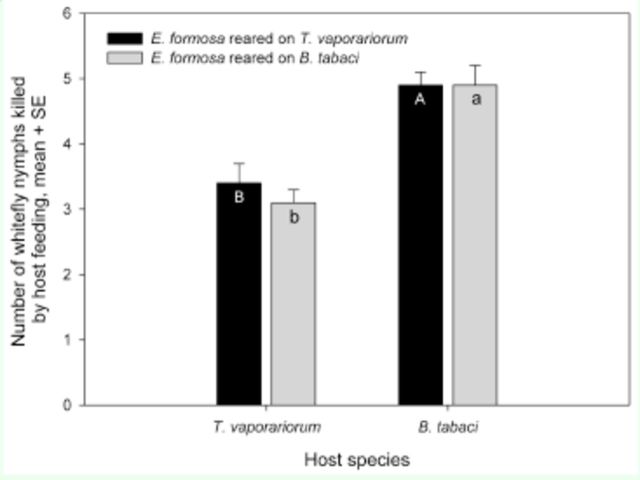
Mean number of *Trialeurodes vaporariorum* or *Bemisia tabaci* nymphs killed in 48 h through host feeding by *Encarsia formosa* reared on nymphs of *T. vaporariorum* or *B. tabaci* . Different upper-case letters in the black bars indicate significant differences in mean number of *T. vaporariorum* nymphs attacked. Different lowercase letters in the grey bars indicate significant differences in mean number of *B. tabaci* nymphs attacked. The paired bars with an * indicate significant differences in mean number of whitefly nymphs between *B. tabaci* and *T. vaporariorum* at *P* < 0.05.

The rearing host species and the interaction of rearing host species*host species attacked had no significant effect on the host feeding of *E. formosa* (rearing host species, F1 96 = 0.44, *P* = 0.5085; rearing host species*host species attacked, F1 96 = 0.44, *P* = 0.5085). However, the host species attacked had a significant effect on host feeding (F1 96 = 48.55, *P* < 0.0001). There was no difference in the number of whitefly nymphs killed by host feeding between the *E. formosa* reared on *T. vaporariorum* and those reared on *B. tabaci* whether *T. vaporariorum* or *B. tabaci* were offered as hosts ( Fig. 2 ). However, the *E. formosa* reared on different host species exhibited different host-feeding capacities on the third instars of *B. tabaci* and *T. vaporariorum* . Regardless of the species on which it was reared, *E. formosa* fed significantly more on the *B. tabaci* nymphs than on the *T. vaporariorum* nymphs ( Fig. 2 ).

### Parasitism.

**Figure 3.**
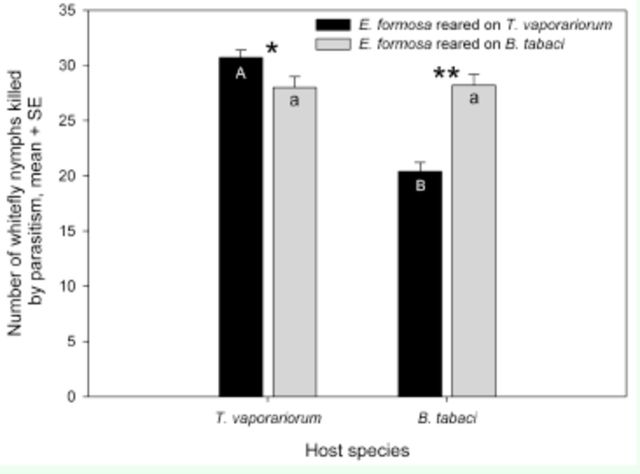
Mean number of *Trialeurodes vaporariorum* or *Bemisia tabaci* nymphs parasitized in 48 h by *Encarsia formosa* reared on nymphs of *T. vaporariorum* or *B. tabaci.* Different upper-case letters in the black bars indicate significant differences in mean number of *T. vaporariorum* nymphs attacked. Different lowercase letters in the grey bars indicate significant differences in mean number of *B. tabaci* nymphs attacked. The paired bars with an * or ** indicate significant differences in mean number of whitefly nymphs between *B. tabaci* and *T. vaporariorum* at *P* < 0.05 or *P* < 0.01, respectively.

The rearing host species, the host species attacked, and the rearing host species*host species attacked interaction had a significant effect on the parasitism by *E. formosa* (rearing host species: F _1,__96_ = 32.38, *P* < 0.0001; host species attacked: F _1,__96_ = 8.62, *P* = 0.0042; rearing host species*host species attacked: F _1,__96_ = 35.53, *P* < 0.0001). The number of whitefly nymphs killed by *E. formosa* through parasitism differed between the *E. formosa* reared on *T. vaporariorum* and those reared on *B. tabaci* depending on which whitefly species were offered as hosts. If *T. vaporariorum* was used as the host, the wasps reared on *T. vaporariorum* parasitized more whiteflies than those reared on *B. tabaci* . If *B. tabaci* was used as the host, however, the wasps reared on *B. tabaci* parasitized more whiteflies than those reared on *T. vaporariorum* ( Fig. 3 ). In addition, the *E. formosa* reared on different host species exhibited different parasitism capacities on the third instars of *B. tabaci* and *T. vaporariorum* . The wasps reared on *T. vaporariorum* parasitized significantly more on *T. vaporariorum* than on *B. tabaci* . However, the wasps reared on *B. tabaci* parasitized similar number of whiteflies on both host species ( Fig. 3 ).

### Total mortality.

**Figure 4.**
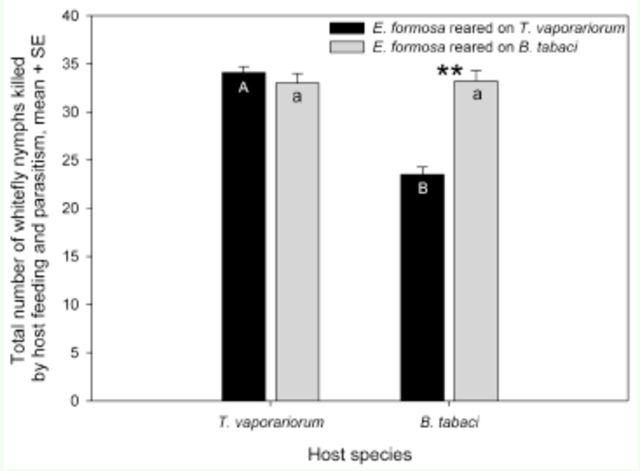
Mean number of total *Trialeurodes vaporariorum* or *Bemisia tabaci* mortality in 48 h caused by *Encarsia formosa* reared on nymphs of *T. vaporariorum* or *B. tabaci.* Different upper-case letters in the black bars indicate significant differences in mean number of *T. vaporariorum* nymphs attacked. Different lowercase letters in the grey bars indicate significant differences in mean number of *B. tabaci* nymphs attacked. The paired bars with an ** indicate significant differences in mean number of whitefly nymphs between *B. tabaci* and *T. vaporariorum* at *P* < 0.01.

The rearing host species, the host species attacked, and the rearing host species*host species attacked interaction had a significant effect on the total mortality of whitefly nymphs caused by *E. formosa* (rearing host species: F _1,__96_ = 32.68, *P* < 0.0001; host species attacked: F _1,__96_ = 22.14, *P* < 0.0001; rearing host species*host species attacked: F _1,__96_ = 35.77, *P* < 0.0001). The total number of whiteflies killed by host feeding and parasitism differed between the *E. formosa* reared on *T. vaporariorum* and those reared on *B. tabaci* depending on which whitefly species was offered as hosts. If *T. vaporariorum* was used as the host, the wasps reared on *T. vaporariorum* and *B. tabaci* caused similar numbers of whitefly mortality through parasitism and host feeding. If *B. tabaci* was used as the host, however, the wasps reared on *B. tabaci* killed significantly more whiteflies than those reared on *T. vaporariorum* ( Fig. 4 ). In addition, the wasps reared on *T. vaporariorum* caused more whitefly mortality on *T. vaporariorum* through parasitism and host feeding than on *B. tabaci* . However, the wasps reared on *B. tabaci* caused similar whitefly mortality on both host species ( Fig. 4 ).

The *E. formosa* adults that emerged from *T. vaporariorum* were significantly larger than those that emerged from *B. tabaci* (head width: *t* = 12.1467, df = 98, *P* < 0.0001; body length: *t* = 6.9665, df = 98, *P* < 0.0001; Table 1 ). Similarly, the hind tibia of the *E. formosa* adults that emerged from *T. vaporariorum* were significantly longer than those that emerged from *B. tabaci* ( *t* = 11.7687, df = 98, *P* < 0.0001).

**Table 1:**
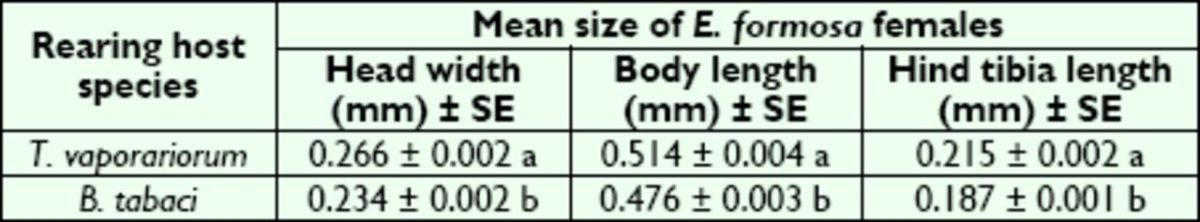
Size comparisons between *Encarsia formosa* reared on *Trialeu-rodes vaporariorum* and those reared on *Bemisia tabaci* .

Means in the same column followed by different letters differ significantly ( *P* < 0.05) by a Student *t* -testBody size of *E. formosa* reared on different host species

## Discussion

Host feeding by the females of hymenopteran parasitoids has been recognized as an asset in biological pest suppression since the mid-1980s ( Jervis and Kidd 1986 ). Some parasitoid species exhibit a strong host-feeding capacity, for example, *Metaphycus helvolus* (Compere) ( DeBach 1943 ), *Trichogramma turkestanica* Meyer ( Hansen and Jensen 2002 ), and *Encarsia sophia* (Girault and Dodd) ( Zang and Liu 2008) , and the effectiveness of their host feeding to control insect pests is similar to or greater than that of their parasitism. Many factors affect the host-feeding behavior of parasitoids, such as environmental variables ( Hansen and Jensen 2002 ), egg load ( Collier 1995 ), host stage, and host density ( Videllet et al. 1997 , Rosenheim and Rosen 1992 ). Earlier Zang and Liu (2009 , 2010) found that the duration of food-deprivation of whitefly parasitoids before their release affected their host-feeding capacity. In addition, the mating status of whitefly parasitoids before their release affected their host-feeding capacity ( Zang et al. 2011b ). In this study, both populations of *E. formosa* reared on *T. vaporariorum* and *B. tabaci* fed more on *B. tabaci* than on *T. vaporariorum* . The rearing host species, however, did not influence host feeding on a particular host. The results indicated that the host-feeding capacity of *E. formosa* was affected by the attacked host species rather than by the host species used for rearing.

Many studies have assessed the effect of the rearing host species on the reproductive performance and host selection behavior of Hymenopteran parasitoids. Generally, the reared host species affects the parasitoid quality and its ability to parasitize target hosts. In *Trichogramma* , *T. pretiosum* Riley reared on *Ephestia kuehniella* Zeller parasitized more eggs of *E. kuehniella* or *Sitotroga cerealella* (Olivier) than those reared on *S. cerealella* ( Lewis et al. 1976 ). The individuals of *T. minutum* reared on *Choristoneura fumiferana* (Clemens), *Lambdina fiscellaria* (Guenee), or *Manduca sexta* (L.) attacked significantly more eggs of *E. kuehniella* than those reared on *E. kuehniella* ( Corrigan and Laing 1994 ). In addition, recent findings by Zang et al. (2011a) indicate that the reproductive performance of the autoparasitoid *E. sophia* can be affected by mating with males originating from different secondary host species. Henter et al. (1996) assessed the variation in the reproductive performance and host selection behavior between populations of *E. formosa* reared on different host species. Their behavior differed in the number of hosts encountered and the reactions to the hosts between the populations of *E. formosa* reared for many years on either *B. tabaci* or *T. vaporariorum* ( Henter et al. 1996 ).

This study indicates that the number of white-fly nymphs parasitized by *E. formosa* differs between the wasps reared on *T. vaporariorum* and those reared on *B. tabaci* depending on which whitefly species was offered as host. In addition, the wasps reared on *T. vaporariorum* parasitized significantly more on *T. vaporariorum* than on *B. tabaci* . The wasps reared on *B. tabaci* , however, parasitized similar numbers of whiteflies on both host species. These results indicate that the parasitism of *E. formosa* is not only affected by the host species attacked but also by the rearing host species.

Hosts vary in quality for oviposition and the host feeding of parasitoids, and host size is the most ubiquitous factor contributing to host quality ( Heimpel and Collier 1996 ). As shown by Zang and Liu (2008) , *E. formosa* , *E. sophia* , and *Eretmocerus melanoscutus* Zolnerowich and Rose feed more on first and second instars (smaller hosts) than on third and fourth instars (larger hosts) of *B. tabaci* . For nymphs of the same stage, *T. vaporariorum* is larger than *B. tabaci* in body size, and *E. formosa* reared on either *T. vaporariorum* or *B. tabaci* fed more on the third instars of *B. tabaci* than on the same stage nymphs of *T.*

*vaporariorum* . *E. formosa* reared on *T. vaporariorum* or *B. tabaci* parasitized significantly more hosts of the whitefly species on which they were reared, which may be a result of long-term adaption to the species. Certainly, the host on which *E. formosa* is reared affects the subsequent host selection either because of a genetic response to selection or as a result of environmental conditioning ( Henter et al. 1996 ).

The larger parasitoids produced from larger hosts, as compared with those from smaller hosts, have exhibited superiority in fecundity and longevity (Hohmann et al. 1988, Corrigan and Laing 1994 ). The individuals of *E. formosa* reared on *T. vaporariorum* are clearly larger than those reared on *B. tabaci* . As expected, *E. formosa* reared on *T. vaporariorum* killed more *T. vaporariorum* nymphs by parasitism than those reared on *B. tabaci* . Additionally, a similar number of *B. tabaci* nymphs were killed by *E. formosa* regardless of the rearing host species. Furthermore, *E. formosa* fed on a similar number of *T. vaporariorum* and *B. tabaci* nymphs regardless of the rearing host species. The host-feeding behavior in parasitoids does not appear to be correlated with the rearing host. In general, the *E. formosa* reared on *T. vaporariorum* exhibit strong biocontrol effectiveness against whiteflies; in this study, they killed more *T. vaporariorum* and a similar number of *B. tabaci* nymphs by parasitism and host feeding than those reared on *B. tabaci* . With the rapid widespread invasion of *B. tabaci* ‘B’ and ‘Q’, currently mixed infestations of *B. tabaci* and *T. vaporariorum* on vegetable crops usually occur in some countries ( Liu et al. 1994 , Arno et al. 2006 , Yan et al. 2011 ). Our results support releasing *E. formosa* mass-reared on *T. vaporariorum* against mixed whitefly infestations in biological control programs.

## References

[R1] Arno J Albajes R. Gabarra R. . 2006 . Within-plant distribution and sampling of single and mixed infestations *of Bemisia tabaci* and *Trialeurodes vaporariorum* (Homoptera: Aleyrodidae) in winter tomato crops . J. Econ. Entomol. 99 : 331 – 340 . 16686130 10.1603/0022-0493-99.2.331

[R2] Burger J. M. S. Kormany A. van Lenteren J. C. v Vet L.E. M. . 2005 . Importance of host feeding for parasitoids that attack honeydew-producing hosts . Entomol. Exp. Appl. 117 : 147 – 154 .

[R3] Collier T. R . 1995 . Host feeding, egg maturation, resorption, and longevity in the parasitoid *Aphytis melinus* (Hymenoptera: Aphelinidae) . Ann. Entomol. Soc. Am. 88 : 206 – 214 .

[R4] Corrigan J. E. Laing. J. E. 1994 . Effects of the rearing host species and the host species attacked on performance by *Trichogramma minutum* Riley (Hymenoptera: Trichogrammatidae) . Environ. Entomol. 23 : 755 – 760 .

[R5] De Barro P. J. Liu S. S. Boykin L. M. Dinsdale A. . 2011 . *Bemisia tabaci:* a statement of species status . Annu. Rev. Entomol. 56 : 1 – 19 . 20690829 10.1146/annurev-ento-112408-085504

[R6] DeBach P . 1943 . The importance of host-feeding by adult parasites in the reduction of host populations . J. Econ. Entomol. 36 : 647 – 658 .

[R7] Giron D Pincebourde S. Casas J. . 2004 . Lifetime gains of host-feeding in a synovigenic parasitic wasp . Physiol. Entomol. 29 : 436 – 442 .

[R8] Hansen L. S. Jensen K. M. V. . 2002 . Effect of temperature on parasitism and host-feeding of *Trichogramma turkestanica* (Hymenoptera: Trichogrammatidae) on *Ephestia kuehniella* (Lepidoptera: Pyralidae) . J. Econ. Entomol. 95 : 50 – 56 . 11942764 10.1603/0022-0493-95.1.50

[R9] Heimpel G. E. Collier. T. R. 1996 . The evolution of host-feeding behavior in insect parasitoids . Biol. Rev. 71 : 373 – 400 .

[R10] Henter H. J. Brasch K. van Lenteren J. C. . 1996 . Variation between laboratory populations of *Encarsia formosa* in their parasitization behavior on the host *Bemisia tabaci* . Exp. Appl. 80 : 435 – 441 .

[R11] Hoddle M Van Driesche R. Sanderson J. . 1998 . Biology and use of the whitefly parasitoid *Encarsia formosa* . v. Entomol. 43 : 645 – 669 . 10.1146/annurev.ento.43.1.645 15012401

[R12] Hoffmann M. P. Ode P. R. Walker D. L. Gardner J. van Nouhuys S. Shelton A. M. . 2001 . Performance of *Trichogramma ostriniae* (Hymenoptera: Trichogrammatidae) reared on factitious hosts, including the target host, *Ostrinia nubilalis* (Lepidoptera : Crambidae) . Biol. Control 21 : 1 – 10 .

[R13] Hohmann C. L. Luck R. F. Oatman E. R. . 1988 . A comparison of longevity and fecundity of adult *Trichogramma platneri* (Hymenoptera: Trichogrammatidae) reared from eggs of the cabbage looper and the angoumois grain moth, with and without access to honey . Entomol . 81 : 1307 – 1312 .

[R14] Jervis M. A. Kidd N. A. C. . 1986 . Host-feeding strategies in hymenopteran parasitoids . Biol. Rev . 61 : 395 – 434 .

[R15] Jervis M. A. Hawkins B. A. Kidd N. A. C. . 1996 . The usefulness of destructive host-feeding parasitoids in classical biological control: theory and observation conflict . Ecol. Entomol . 21 : 41 – 46 .

[R16] Lewis W. J. Nordlund D. A. Gross H. R. Jr. Perkins W. D. Knipling E. F. Voegele J. . 1976 . Production and performance of *Trichogramma* reared on eggs of *Heliothis zea* and other hosts . Environ. Entomol . 5 : 449 – 452 .

[R17] Liu T. X. Oetting R. D. Buntin G. D. . 1994 . Evidence of interspecific competition between *Trialeurodes vaporariorum* (Westwood) and *Bemisia tabaci* (Gennadius) (Homoptera, Aleyrodidae) on some greenhouse-grown plants . J. Entomol. Sci. 29 : 55 – 65 .

[R18] Nell H. W. Sevenster-van der lelie L. A. Woets J. Van Lenteren J. C. . 1976 . Parasite‒host relationship between *Encarsia formosa* (Hymenoptera‒Aphelinidae) and *Trialeurodes vaporariorum* (Homoptera‒Aleyrodidae). Selection of host stages for oviposition and feeding by parasite . J. Appl. Entomol . 81 : 372 – 376 .

[R19] Ozder N Kara G. . 2010 . Comparative biology and life tables of *Trichogramma cacoeciae* , *T. brassicae* and *T. evanescens* (Hymenoptera: Trichogrammatidae) with *Ephestia kuehniella* and *Cadra cautella* (Lepidoptera: Pyralidae) as hosts at three constant temperatures . Biocontrol Sci. Technol. 20 : 245 – 255 .

[R20] Rosenheim J. A. Rosen D. . 1992 . Influence of egg load and host size on host-feeding behavior of the parasitoid *Aphytis lingnanensis* . tomol. 17 : 263 – 272 .

[R21] Roskam M. M. Wessels G. H. van Lenteren J. C. . 1996 . Flight capacity and body size as indicators of quality for the whitefly parasitoid *Encarsia formosa* . ct. Exp. Appl. Entomol. Nether. Entomol. Soc. 7 : 159 – 164 .

[R22] Scopes N. E. A. Biggerstaff S. M. . 1971 . The production, handling and distribution of the whitefly *Trialeurodes vaporariorum* and its parasite *Encarsia formosa* for use in biological control programs in glasshouses . Plant Pathol. 20 : 111 – 116 .

[R23] Shishehbor P Brennan P. A. . 1995 . Parasitism of *Trialeurodes ricini* by *Encarsia formosa:* level of parasitism, development time and mortality on different host plants . Entomophaga 40 : 299 – 305 .

[R24] Tang Q. Y. Zhang. C. X. 2012 . Data Processing System (DPS) software with experimental design, statistical analysis and data mining developed for use in entomological research . Insect Sci. 20 ( 2 ): 254 – 260 . DOI 10.1111/j.1744-7917.2012.01519.x. 23955865 10.1111/j.1744-7917.2012.01519.x

[R25] Ueno T Ueno K. . 2007 . The effects of host-feeding on synovigenic egg development in an endoparasitic wasp, *Itoplectis naranyae* . J. Insect Sci. 7 :46. Available online: www.insectscience.org/7.46 10.1673/031.007.4601 PMC2999447 20345297

[R26] Van Alpen J. J. M. Nell H. W. Sevenster-van der Lelie L. A. . 1976 . The parasite‒host relationship between *Encarsia formosa* Gahan (Hym., Aphelinidae) and *Trialeurodes vaporariorum* Westwood (Hom., Aleyrodidae). The importance of host feeding as a mortality factor in greenhouse whitefly nymphs . IOBC/WPRS Bull . 4 : 165 – 169 .

[R27] Van Roermund H. J. W. Van Lenteren J. C. . 1997 . Analysis of foraging behaviour of the whitefly parasitoid *Encarsia formosa* on a plant: A simulation study . Biocontrol Sci. Technol . 7 : 131 – 151 .

[R28] Videllet P Albajes R. Gabarra R. . 1997 . Host-feeding activity of *Encarsia pergandiella* Howard on *Bemisia tabaci* (Gennadius) . Bull. OILB/SROP 20 : 147 – 152 .

[R29] Yamamura N Yano E. . 1988 . A simple model of host‒parasitoid interaction with host-feeding . Res. Popul. Ecol . 30 : 353 – 369 .

[R30] Yan Y Peng L. Liu L. W. Wan F. H. Harris M. K. . 2011 . Host plant effects on alkaline phosphatase activity in the whiteflies, *Bemisia tabaci* Biotype B and *Trialeurodes vaporariorum* . J. Insect Sci . 11 :9. Available online: www.insectscience.org/11.9 10.1673/031.011.0109 PMC3281299 21521136

[R31] Zang L. S. Liu. T. X. 2008 . Host feeding of three whitefly parasitoid species on *Bemisia tabaci* B biotype, with implication for whitefly biological control . Entomol. Exp. Appl. 127 : 55 – 63 .

[R32] Zang L. S. Liu. T. X. 2009 . Food-deprived host-feeding parasitoids kill more pest insects . Biocontrol Sci. Technol . 19 : 573 – 583 .

[R33] Zang L. S. Liu. T. X. 2010 . Effects of food deprivation on host feeding and parasitism of whitefly parasitoids . Environ. Entomol. 39 : 912 – 918 . 20550806 10.1603/EN09266

[R34] Zang L. S. Liu T. X. Wan F. . 2011a . Reevaluation of the value of autoparasitoids in biological control . PLoS ONE 6 : e20324. doi: 10.1371/journal.pone.0020324. 10.1371/journal.pone.0020324 PMC3102091 21633501

[R35] Zang L. S. Liu T. X. Zhang F Shi S. S. Wan F. H. . 2011b . Effects of mating status on host feeding and parasitism in two whitefly parasitoids . Insect Sci . 18 : 78 – 83 .

